# Participatory Methods to Support Climate Adaptation for Older Adults Living in Vulnerable Urban Areas: An Ethnographic Study

**DOI:** 10.3390/ijerph22060850

**Published:** 2025-05-29

**Authors:** Joel Bruno da Silva, Bibiana Tini, Ana Martins, Inês Mimoso, Teodora Figueiredo, Ana Silva Fernandes, Franklin Gaspar, Gisela Lameira, Luís Midão, Leovaldo Alcântara, Md Imtiaz Ahmad, Luísa Batista, Pedro Rocha, Rui Jorge Garcia Ramos, Sara Cruz, Cecília Rocha, Helena Corvacho, Anabela Ribeiro, Paulo Conceição, Fernando Alves, Elísio Costa

**Affiliations:** 1 RISE—Health, Competence Center for Active and Healthy Ageing, Faculty of Pharmacy, University of Porto, Rua Jorge de Viterbo Ferreira 228, 4050-313 Porto, Portugal; jbruno@ff.up.pt (J.B.d.S.); ifmimoso@ff.up.pt (I.M.); tgfigueiredo@ff.up.pt (T.F.); lmidao@ff.up.pt (L.M.); up202001410@edu.icbas.up.pt (L.A.); 2 CITTA—Research Centre for Territory, Transport and Environment, Faculty of Engineering, University of Porto, 4200-465 Porto, Portugal; up202300547@edu.fe.up.pt (B.T.); up202204098@edu.fe.up.pt (F.G.); up202202072@edu.fe.up.pt (M.I.A.); lbatista@fe.up.pt (L.B.); scruz@fe.up.pt (S.C.); carocha@fe.up.pt (C.R.); psc@fe.up.pt (P.C.); alves@fe.up.pt (F.A.); 3 CEAU—Center for Studies in Architecture and Urbanism, Faculty of Architecture, University of Porto, 4150-564 Porto, Portugal; amartins@arq.up.pt (A.M.); alfernandes@arq.up.pt (A.S.F.); glameira@arq.up.pt (G.L.); rramos@arq.up.pt (R.J.G.R.); 4 School of Medicine and Biomedical Sciences, University of Porto (ICBAS-UP), Rua de Jorge Viterbo Ferreira, 228, 4050-313 Porto, Portugal; parocha@icbas.up.pt; 5 CONSTRUCT—Institute of R&D in Structures and Construction, Faculty of Engineering, University of Porto, 4200-465 Porto, Portugal; corvacho@fe.up.pt; 6 CITTA—Research Centre for Territory, Transport and Environment, Department of Civil Engineering of the University of Coimbra, 3030-788 Coimbra, Portugal; anabela@dec.uc.pt

**Keywords:** climate adaptation, older adults, ethnographic studies, vulnerable urban areas, participatory methods, inclusive and responsive urban design and planning

## Abstract

Urban environments and climate-related challenges impact older adults’ health and well-being. To address these challenges, climate adaptation strategies and urban design guidelines should be tailored to older adults’ needs. Ethnographic studies can help identify these needs by involving them directly in the research process. This study uses ethnographic research to explore older adults’ perceptions and behaviours regarding climate change risks and impacts, health, and mobility challenges in a vulnerable urban area—São Roque da Lameira, Porto, Portugal. It studies the applicability and complementarity of four participatory methods that can inform urban design: (I) semi-structured interviews, (II) ‘go-along’ interviews, (III) user observations, and (IV) emotional mapping. The qualitative data collected were analysed through thematic and spatial analysis. Common themes emerged between the four methods, including concerns about accessibility, safety, and comfort, such as uneven pavements, lack of seating, and poor infrastructure for people with reduced mobility. Participants recommended improvements, such as more green spaces and better pedestrian infrastructure quality. Notably, each method uncovered distinct dimensions, highlighting the added value of a multi-method approach. This study demonstrates that combining participatory methods offers deeper, context-specific insights to inform age-friendly and climate-resilient urban design. Future research should take climate-focused methods and a multidisciplinary approach into consideration.

## 1. Introduction

The world’s population is ageing, and the proportion of people over the age of 65 is on the rise, particularly in developed countries [1]. This demographic shift is accompanied by common declines in physical and mental capacity. While some health variations among older adults are genetic, the majority result from their physical and social environments, as well as personal behaviours and daily routines [2].

The importance of the neighbourhood’s physical and social environment for older adults’ healthy ageing and well-being has long been recognised. Physical environments influence health both directly and by shaping opportunities, safety, decisions, and behaviours. Supportive environments, including universal accessibility to public buildings, transport modes, and pedestrian areas, help older adults maintain activities that are important to them, even with limited capacities. Neighbourhood features significantly impact older adults’ ability to stay mobile, active, and socially engaged [2,3].

A recent systematic review examining the influence of urban neighbourhood attributes on the well-being of older adults found that natural spaces are frequently associated with positive well-being outcomes. Other factors positively linked to well-being included transportation options, urban amenities, and access to healthcare. Neighbourhoods that incorporate these features may better support well-being among older adults [4]. It was also reported that pedestrian infrastructure, safety, facility access, aesthetics, and environmental conditions significantly influence physical activity among older adults [5]. Another literature review identified various neighbourhood environmental factors linked to health outcomes in later life, such as higher population density and rurality, walkability and street connectivity, access to services and amenities, neighbourhood quality and disorder, as well as the presence of parks and green, blue, or open spaces [6].

To create sustainable urban environments extensive to the older adults and extensive to the total population, urban planners and architects must consider local and global climate factors and climate change in both current and future city design [7]. Research in this field primarily focuses on issues, such as urban heat islands, low outdoor thermal comfort, and compromised air quality, alongside urban planning strategies to mitigate these climatic challenges [8,9,10]. However, despite substantial evidence on the climatic impacts of urbanisation, effectively incorporating this knowledge into practical development remains challenging. This can be attributed to methods available during early design stages [11].

This concern is heightened in regions where urban climate-related phenomena are more intense, and the population of older adults is high. In fact, a recent systematic review showed that climate change is associated with an increased risk of mortality and morbidity outcomes in older adults, including cardiovascular, respiratory, renal and mental diseases, as well as physical injuries. Additionally, gender, socioeconomic status, and education level are factors that influence vulnerability to these effects [12].

Urban vulnerability is understood not only as a condition of individuals but as a characteristic of the spaces they inhabit. The intersection of climate exposure, adaptive capacity, and population resilience defines how urban environments can themselves become vulnerable [13]. In this context, older adults experience heightened risks not solely due to personal health or mobility limitations but because they are embedded in territories with inadequate infrastructure, limited adaptation capacity, and greater exposure to climatic challenges. The condition of these spaces directly affects their well-being, amplifying pre-existing inequalities and making targeted urban interventions essential for mitigating these risks.

This is the motivation behind the “Climate Adaptation For Older People Living In Vulnerable Urban Areas: Designing a Climate-Responsive and Community-Based Methodology—CAOP” project (REF. PTDC/GES-URB/2038/2021). This project seeks to address and mitigate the adverse effects of climate change on older adults in vulnerable urban areas. To achieve this, initiatives were adopted that encouraged the participation of older adults living in these areas, aiming to understand how they experience the impacts of climate change based on their experiences and perspectives on the issue and its consequences.

The CAOP project aligns with a broader shift in urban planning, where public participation plays an increasingly crucial role, marking a significant change in the development of modern cities. Citizens, through the sharing of their experiences and perspectives, now play an active role in shaping urban projects and public policies, thereby reinforcing their importance in transforming the spaces they inhabit. This process of public participation can take various forms depending on the level of involvement and influence in decision-making. Participatory planning is based on key principles, such as recognising societal diversity, fostering dialogue to understand different perspectives, and ensuring no individual or group is excluded from the decision-making process. It emphasises collaborative interaction, mutual learning, and the creation of locally developed standards, aiming for a more inclusive and democratic urban transformation [14].

Within the activities of CAOP project, this research employs an ethnographic approach —defined as immersive engagement within a community and the collection of descriptive data through fieldwork [15], to examine the perceptions of older adults regarding climate change and its impact on health, as well as their experiences with daily life, health, and mobility challenges in a vulnerable urban area. Additionally, it explores the applicability and complementarity of four participatory methods that can help urban design, assessing their strengths and limitations in addressing the specific needs of this population group. By comparing and discussing these approaches, the paper highlights the role of ethnographic research in informing inclusive urban design. The study provides context-specific insights into how older adults experience environmental and climate-related challenges, supporting more inclusive adaptation strategies and fostering healthy ageing and ageing-in-place. The research team comprised a multidisciplinary group of experts with backgrounds in health and ageing, education, participatory methods and tools, design, architecture, climate adaptation, urban planning, and engineering. This diversity of expertise enabled the integration of multiple perspectives, ensuring that the participatory process implemented was both inclusive and contextually relevant.

## 2. Materials and Methods

### 2.1. Research Design

This study adopts a qualitative phenomenological approach to explore the lived experiences and perceptions of older adults regarding the impacts of climate change on health, mobility, and daily life in a vulnerable urban area [16,17]. Drawing on this framework, an ethnographic research design was applied, using four participatory methods: individual semi-structured open-ended in-depth interviews (I), go-along interviews (II), user observations (III), and emotional mapping (IV). The study explores the application and combination of these participatory methods to assess their strengths and limitations in addressing the specific insights of this population group. The selection of methods considered their applicability and practical effectiveness, particularly in engaging older adults in meaningful and inclusive ways.

(I) Open-ended in-depth interviews explored participants’ perceptions and experiences of climate change and the urban environment, focusing specifically on health, mobility, and well-being. This method was chosen for its ability to elicit rich, nuanced narratives on lived experience and personal values. It is particularly suited to exploring complex and sensitive topics such as ageing and health [18].

(II) Go-along interviews examined participants’ real-time interactions with public spaces, paying attention to both “spaces to stay” and “spaces to go to” [19]. This method provided valuable insights into how older adults engage with their surroundings and contributed to understanding the spatial organisation and functionality of the urban environment.

(III) Direct observations were conducted in various public spaces to understand how these areas were used on hot summer days. Observations focused on climatic conditions, user behaviours and preferences, and possible health-related perceptions influencing choices of routes, meeting points, and places to stay. This method offered an objective perspective on spatial practices and environmental interaction, complementing the subjective accounts gathered through interviews [20]

(IV) Emotional mapping aimed to identify positive and negative aspects of selected public spaces and how these could be improved to better support the needs of older adults in the context of climate change. This method elicited subjective and affective responses and was especially suitable for small-group settings, fostering dialogue on accessibility, safety, maintenance, and comfort [21].

### 2.2. Case Study Area

The study was conducted in São Roque da Lameira, an urban area located in Campanhã, the easternmost parish of the city of Porto, Portugal, with approximately 46 hectares. The area is located within the designated Urban Rehabilitation Areas (ARU)—the ARU of *Corujeira*. An ARU is a geographically defined area where, due to building insufficiency, deterioration, or obsolescence of buildings, infrastructure, equipment for collective use, and urban and green collective spaces, particularly regarding their usability, structural integrity, safety, aesthetics, or health standards, an integrated intervention is warranted [22].

In this context, São Roque da Lameira presents social–spatial vulnerabilities related to its physical and demographic structure, exacerbated by the peripheral location within Porto. This area includes public housing and precarious housing developments (‘ilhas’), and it is bordered by physical barriers, namely a railway line and an intensive traffic road network. The area includes facilities (e.g., schools and social centre), vacant, and expectant spaces corresponding to obsolete industrial areas, and a deficit in public space [23]. Additionally, the dense urban development and topography, the slight elevation compared to contiguous areas to the west, and the industrial and road network materials increase heat accumulation during the day and extend its retention [24]. These factors, along with other environmental conditions (e.g, air pollution), may contribute to heightened climatic vulnerability over time. Furthermore, it is one of the most aged areas in the ARU, with 31.0% of the population over 65 years old in 2021 [25].

São Roque da Lameira has different morphological characteristics, accommodating a historic main road, São Roque da Lameira Street, and one large residential area, São Roque da Lameira neighbourhood, with a group of multifamily housing buildings (Grupo de Moradias Populares, 1959–1962), and a layout of single-family houses with private courtyards (Bairro de Casas Económicas, 1939–1943), placed around a tree-lined square (Largo de Valverde), and surrounded by narrow streets and sidewalks restricting mobility and urban enhancements. São Roque da Lameira Street remains a high-traffic corridor with public transport and local services. However, old infrastructure, dense urban activity, and heavy traffic pose challenges to walkability, particularly for older residents. While the street serves multiple functions, the neighbourhoods are primarily residential, highlighting a contrast between mixed-use and monofunctional urban spaces.

**Figure 1:**
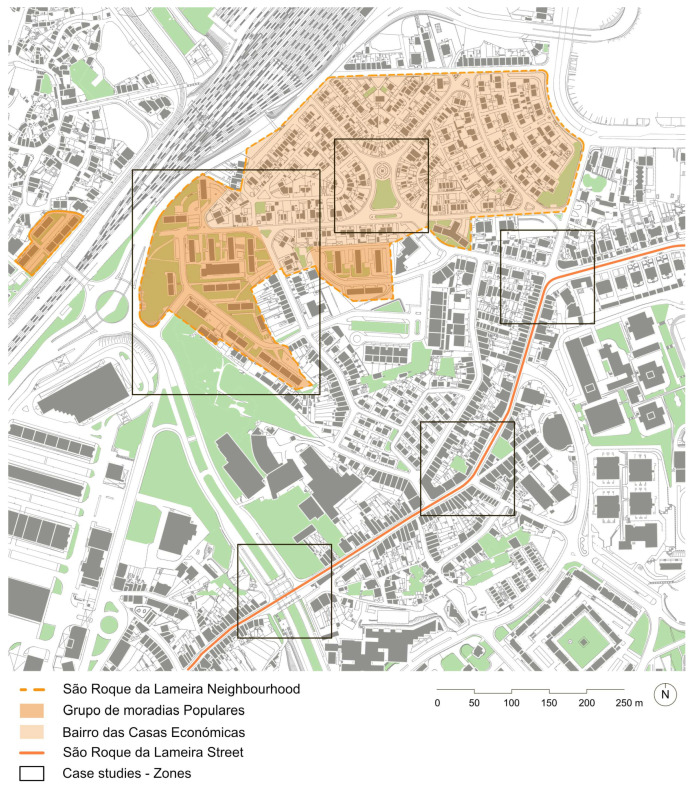
São Roque da Lameira area and case studies. Source: CMP, © CAOP.

The São Roque da Lameira area illustrates the socioenvironmental conditions and morphological characteristics encompassing a diverse population. It reflects the area’s ageing demographics, health challenges, social disparities, and physical constraints. Addressing these challenges requires comprehensive public space design strategies, improved accessibility to health services, and community support initiatives to improve the quality of life for the ageing population. To define these strategies, as examples, public spaces were selected in São Roque da Lameira Street and neighbourhood (Figure 1).

### 2.3. Setting and Participants

Table 1 shows the main aspects of the four participatory methods: individual semi-structured open-ended in-depth interviews (I), ‘go-along’ interviews (II), user observations (III), and emotional mapping (IV). All the participants were 65 years or older.

(I) For the open-ended in-depth interviews, a local daycare centre within the study area agreed to participate. The contact person in the daycare centre identified and invited participants who complied with the study criteria, allowing for purposive sampling to select the interviewees [26]. All interviews were conducted at the daycare centre. To participate in the study, individuals were required to reside in or have extensive knowledge of the São Roque da Lameira neighbourhood and street (1) and to be able to communicate effectively in Portuguese (2). Interviews were conducted entirely in Portuguese, the participants’ native language as well as that of the interviewers. Through purposive sampling, 16 participants were recruited. Thematic saturation was used as the criterion to stop data collection. Saturation was reached when coding analysis revealed consistent repetition of key themes and no emergence of novel categories, indicating that data sufficiency had been achieved. Two factors were important for thematic saturation: the homogeneity of the sample—older adults from the same neighbourhood with shared experiences and the objectivity of the topic. Saturation was achieved with a relatively small number of interviews, in line with established qualitative research practices [27]. Each participant participated in one individual semi-structured interview.

(II) The ‘go-along’ interviews were conducted in open/green spaces within the São Roque da Lameira neighbourhood and in crossing or intersection spaces along São Roque da Lameira Street. These locations were previously selected due to their relevance in shaping the residents’ daily experiences and interactions with the urban environment. Regarding the participants, they were conveniently approached within the study area and consisted exclusively of residents of the São Roque da Lameira neighbourhood. A total of 36 individual semi-structured interviews were conducted, allowing for an in-depth exploration of the participants’ perceptions regarding sense of belonging and well-being, comfort and safety, climatic factors (heat and wind), accessibility, amenities, urban furniture, and public space infrastructure. Sampling was also discontinued upon reaching thematic redundancy. The semi-structured ‘go-along’ interviews enabled researchers to capture participants’ lived experiences as they moved through their everyday routines, facilitating richer insights into their relationship with public space.

(III) The direct observations of users’ behaviours captured the patterns of movement and gathering in the main public spaces of the case study area, within a context of heat, to realise needs and preferences in the spatial appropriation (taking into account aspects of diversity, such as age, gender, and culture), as well as the current exposure to heat incidence. Nine open/green spaces were observed, six in the neighbourhood and three on the street, according to existing important squares in the area.

(IV) Emotional mapping was designed to ensure inclusivity, given the physical limitations of the target population, while fostering meaningful dialogue about accessibility, maintenance, health, and the potential for prolonged use of public spaces. In the emotional mapping activity, nine participants were consulted. To capture a comprehensive understanding of the area, the research team initially conducted a thorough walkthrough of the study zone, photographing key locations. For the participatory activity, 2–3 representative images were selected from each sub-area (e.g., São Roque da Lameira neighbourhood and street, squares, main streets) within the study zone. These images were chosen to reflect the diversity of the urban environment and its specific vulnerabilities. During the activity, participants were shown the selected images and asked to express their perceptions and emotions using red and green cards. A red card indicated a negative perception (e.g., poor accessibility, inadequate maintenance, or health concerns), while a green card signified a positive perception (e.g., good accessibility, well-maintained spaces, or health-promoting features). Prior to the activity, participants were briefed on the purpose of the cards and provided with examples of scenarios that might warrant a red or green response. Importantly, the activity was not limited to card selection; after each image, participants engaged in discussions to elaborate on their choices. These conversations explored their reasoning, suggestions for improvement, and whether they felt comfortable and safe in the depicted spaces.

### 2.4. Data Collection

(I) For the open-ended interviews, data were collected over a four-month period (June to October 2024) during three visits to the daycare centre. A total of 15 interviews were conducted. At the outset of each interview, the research team provided participants with detailed information, including the purpose and scope of the study and the approximate interview duration (60 min). During the interviews, audio recordings were made to capture the discussions in detail. After the interviews, field notes were made. These interviews were later transcribed.

**Figure 2:**
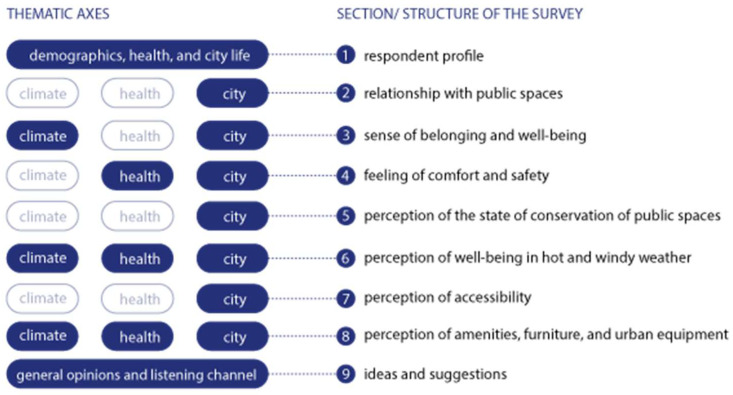
Diagram of the structure of the ‘go-along’ interviews. Source: Created by the authors (2025).

The guide for the interviews (see Appendix A) was developed based on existing knowledge and a comprehensive literature review. All members of the research team collaboratively refined the questions. The guide was piloted with two individuals who shared similar characteristics with the study participants to ensure clarity and comprehension. None of the individuals involved in the piloting participated in the actual study. Following this pilot, adjustments were made to the guiding questions to enhance clarity and relevance.

(II) The ‘go-along’ interviews involved individuals who were guided through two areas—the São Roque da Lameira neighbourhood and street—where they live and regularly walk to reach specific destinations. During these interviews, participants were asked a pre-set list of questions (see Appendix B) focused on thematic axes: demographics, health, urban life, climate, and general opinions. The main objective was to collect information about the lives and experiences of older people in public spaces. The survey was organised into blocks of interrelated questions, directly linked to the fields of climate, health, and the city (Figure 2).

In total, 36 interviews were conducted in the study area over 9 days (non-consecutive), strategically distributed to capture a wide variety of experiences, taking into account the different times of day and weather conditions. Pedestrians on these trips and stops were randomly approached by the researchers in January and February 2025 from 9:00 to 10:00 a.m., 12:30 to 13:30 p.m. and 16:00 to 17:00 p.m.

At the beginning of each interview, two members of the research team carefully explained the study to the participants to ensure clarity and transparency. They provided detailed information about the aim and significance of the research, stressing the importance of the participants’ contributions to understanding the challenges and opportunities related to the transformation of the *São Roque* area. In addition, the interviewers indicated the expected duration of the interview (around 15 min) in order to set expectations.

There was no pre-selection of routes, but it was crucial to ensure that participants engaged in meaningful and relevant places that reflected their experiences of the public spaces of the neighbourhood and the street of *São Roque da Lameira*. As participants walked, they were encouraged to comment on specific environmental and urban design features, such as the availability of shaded and seating areas or green infrastructure.

(III) For the user observations, the data collection process was undertaken through fieldwork conducted during the month of August 2024 across six visits to the *São Roque da Lameira* neighbourhood and Street, throughout a sequence of hot summer days (with temperatures between 20.05 °C and 28.10 °C). The observations were held twice a day, at late mornings and afternoons, 10:00 am–1:30 pm and 5:00–8:00 pm, in order to study both the period of midday, hours of intense heat, (to capture grocery-shopping routines, lunch breaks, social gathering, during hot weather), as well as the finishing periods of school and work (to see parenting roles, family logistics, and home return trajectories). The selection of these target periods was derived from longer observations of these areas and the recognition of these schedules as the ones with more movement and of strategic relation with the heat exposure. The six spaces in the neighbourhood and three on the street were observed for a period of 30 min each, on workdays and weekends. The study mapped the trajectories or staying locations of the users according to their age and gender, resulting in a cartography of gathering spaces, resting points and mobility patterns, detecting difficulties and preferences. A targeted approach was employed to ensure consistency and reliability in the observations. The researchers meticulously documented the behaviours and interactions of public space users, noting their physical characteristics, such as age, gender, and group composition. Along with the drawn cartography, the KoBoToolbox version 2024.2.4 was used for mapping and recording the data, facilitating the systematic collection of movement patterns and key behavioural trends. Observations were conducted in pre-selected public spaces that represented a variety of environmental conditions. The researchers specifically focused on the gathering areas, documenting where people congregated outdoors and how weather conditions influenced their activities. Notably, spaces exhibiting clear signs of adaptation or neglect, such as well-maintained green spaces with seating versus areas with deteriorating infrastructure, were carefully noted. This was done to investigate potential connections between urban renewal efforts and climate adaptation strategies. By selecting public spaces that illustrated both challenges and opportunities, the observations provided a comprehensive foundation for the study. The users did not know they were being observed, so as not to interfere with the behaviours and their intuitive interaction with the space. This approach ensured that the findings would be relevant to the design of inclusive, sustainable urban spaces that support health, well-being, and climate resilience.

(IV) For the emotional mapping activity, data were collected during a single session held in February 2024 at a local daycare centre. The session lasted approximately 60 min and involved nine participants. At the beginning of the session, the research team provided participants with detailed information about the study, including the purpose, scope, and expected duration of the activity. No audio or visual recordings were made during the session. Instead, the research team took detailed field notes to capture the discussions and responses.

In total, 61 older residents participated in the study.

### 2.5. Data Processing and Analysis

(I) Thematic analysis was used for open-ended interviews, as a qualitative methodology for analysing the data collected. This methodology followed the concepts, definitions and six-step framework already described extensively elsewhere [28], which was based on the approach of Braun and Clarke [29]. Briefly, this process began with familiarisation with the data, including transcription of audio recordings and review of field notes, both of which were translated into English (i). Initial codes were then generated, reflecting key topics raised by participants (ii), which were grouped into themes, such as health, mobility, perceptions of comfort and safety in public space, and the impact of climate and extreme weather events (iii). These themes were reviewed for coherence and consistency (iv), clearly defined and named (v), and synthesised into a narrative report aligned with the study’s objectives (vi).

(II) The ‘go-along’ data interviews were conducted in a sequential three-step process, starting with the transcription of field notes (i). The following step, data collection, was organised using a thematic approach defined during the creation of the questionnaire using LimeSurvey Community Edition version 6.14.0+250520. This was supported by the work of Kusenbach and Holgersson [30,31] (ii), which framed the key themes explored during the ‘go-along’ interviews. However, an additional subject on climate adaptation was introduced, exploring perceptions of climate change and its health impacts. The results, organised in an Excel spreadsheet (iii), allowed for segmented reading by evaluation zones, enabling each piece of data to be analysed in relation to the questions asked at each location. This is justified by the fact that each point of approach had specific characteristics, allowing both an isolated analysis of opinions and a cross-sectional view, including the gender perspective. Based on these results, many of which were obtained through open-ended questions, the responses were categorised. This process differentiated questions that could be reformulated to include closed alternatives from the ones requiring the support of complementary participatory methods to achieve greater precision in capturing the opinions of the participants. Finally, the findings showed the potential of combining diagrams/graphs and textual analysis, providing a more comprehensive interpretation of the data. The diagrams/graphs offered a visual representation of the main patterns, relationships and themes, making complex information easier to understand. Textual analysis complemented this visualisation by providing detailed explanations, contextualising the findings, and highlighting nuances that might not be immediately apparent graphically.

(III) The user observations were useful for understanding how different age groups engaged with the public space. Through direct observations, the study monitored the movement of people within the São Roque da Lameira area, noting their social behaviours, preferred gathering spots, and how they interacted with features, like benches, sidewalks, and crossings. These observations highlighted patterns, such as older adults’ preference for quieter, shaded areas, as well as the movement patterns of pedestrians. To support this, a methodology was developed in three steps. Firstly, several materials were prepared, including Geographical Information Systems (GIS) Field Maps elaborated in Qgis Software and a questionnaire in KoBoToolbox supported on the Project for Public Space (PPS) studies [32] (i). Photographs, hand-drawn sketches for spatial mapping, field notes, and questionnaires filled out by researchers were then used to visualise these behaviours. This process included creating maps to identify high-traffic zones, mapping pedestrian trajectories, and pinpointing preferred spaces for staying or social interaction (ii). Additionally, the data were included in GIS to facilitate further analysis, mainly related to heat stress. By combining these qualitative tools, the study identified design opportunities to improve accessibility, enhance social spaces, and ensure the space addressed the diverse needs of all users, with a particular focus on older adults (iii).

(IV) Emotional mapping employed thematic analysis, following Braun and Clarke’s framework [29], to analyse data collected with the participants. Field notes from the session were reviewed, and initial codes were generated (i–ii). These codes were grouped into three main themes: barriers, facilitators, and improvement suggestions (iii). Themes were refined and reviewed for coherence (iv–v), then synthesised into a narrative report (vi). This approach provided insights into adapting public spaces for older adults, aligning with climate resilience and inclusive design goals.

### 2.6. Ethical Considerations

This study was reviewed and approved by the ethics committee and the data protection officer of the University of Porto (Report n° 6 June 2023 and Report n° R-26/2025/10 March 2025). In the open-ended interviews (I), “go-along” interviews (II) and emotional mapping (IV), Participants were informed that their involvement was entirely voluntary and confidential and that they could withdraw at any time without any consequences. Informed consent was obtained through a signed document, which was countersigned by a research team member to confirm that all relevant information had been effectively communicated.

## 3. Results

The results section is organised based on the four participatory methods used in this study. This section will present information on the analyses and results found in each method. The data presented, rather than aiming for statistical generalisation, focuses on how participants interpret, feel, and respond to their neighbourhood’s physical and social characteristics in the context of climate change.

(I) Open-ended interviews

Sample Description

This study had a sample of 16 participants (Table 2), 12 female and 4 male, with an average age of 81.6 ± 8.6 years, reflecting a gender imbalance typical of this age group. Concerning the participants’ level of education, the majority had attended the first cycle (3rd and 4th grade), except two participants who had participated in the second cycle (10th and 11th grade). Regarding their living conditions, it was possible to identify that 11 participants lived alone, and 5 lived with their children and grandchildren.

**Figure A1:**
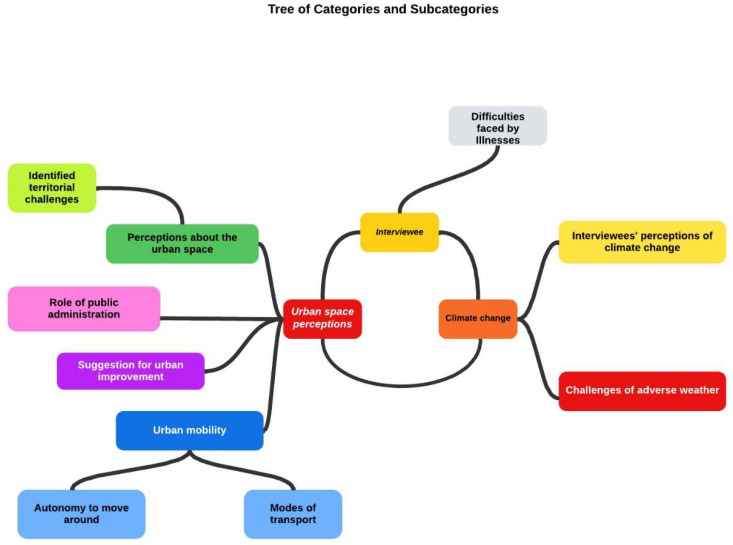
Tree of categories and subcategories of the open-ended interviews. Source: CMP, © CAOP.

Analyses of the interviews with users revealed information about participants’ perceptions, experiences, and expectations of the territory and the subjects covered in the study. After reading and reviewing the content of the interviews, a thematic analysis was conducted, and eight categories and two subcategories were created to interpret the interviewees’ statements and assign meaning to their discourse (Table 3) (see Appendix C: Figure A1). We sought to understand participants’ perceptions of the phenomenon of climate change and the difficulties they experienced in the face of adverse weather conditions. We also identified their perception of the urban space where they live, taking into account the limiting aspects and suggestions for improving the area, with a view to the role of public administration in this process. In addition to these themes, we also sought to recognise users’ perceptions of urban mobility, taking into account the means of transport they use most, the degree of autonomy they have to get around the area, as well as the weaknesses and limitations they face in getting around due to their illnesses.

1. Participants’ perceptions of climate change

One key finding was the significant concern among older adults about increasing temperatures and extreme weather events, which they perceive as direct threats to their health and well-being. Regarding the participants’ perceptions of climate change, it was possible to recognise that some interviewees feel that the phenomenon of climate change has influenced the transformation of the weather, especially the seasons. These participants mention that daytime temperatures are becoming higher, a meteorological characteristic that has spread to other seasons beyond summer. In other words, users realise that there is instability in the weather and, in particular, an increase in temperature. Another link they made was the influence of anthropogenic actions on this phenomenon.

(…) Everything is different. We have uncontrolled weather in all seasons. (…) It is very destabilised. And the disasters, the oceans, the catastrophes, that are increasing more and more (…)

(E15, female, 81 years old)

(…) Yes, there is a difference. The summer season did not last as long as it does now. Now it is very hot. And then we are as well off as we are. There is cold, there is heat. Rapid changes, and that hurts people a bit (…)

(E7, male, 81 years old)

(…) Man has damaged it. He has damaged everything, he wants to do this, he wants to do that, he wants more, he wants to do things he should not do (…)

(E6, woman, 77 years old)

2. Challenges of adverse weather

After identifying participants’ perceptions of climate change, the next step was to understand how these changes affect their daily lives. Some participants reported avoiding outdoor activities during hot weather, limiting their social interactions and physical activity. It was observed that some participants experienced health complications as a result of extreme climate variations. They mention that this variation in climatic conditions has jeopardised the quality of their life and health. In other words, users perceive that adverse weather conditions have aggravated the symptoms of their illnesses.

(…) We always catch colds. Look, this weekend was hot and today is cold. It is very uncertain. I have been coughing a lot as a result of the weather (…)

(E4, woman, 85 years old)

(…) It affects me because I have arthritis in my hips, and it is a pain you cannot imagine. (…) The change of weather for the bones is no joke (…)

(E5, male, 81 years old)

(…) I have been feeling it because I get home and I can’t handle the pain in my bones (…)

(E9, woman, 91 years old)

3. Urban Mobility

### 3.1. Means of Transport

After identifying limitations in participants’ ability to move around their area, the study explored their perceptions of urban mobility, the modes of transport they use, and their level of autonomy in navigating the environment. It was, therefore, possible to identify that participants get around by different means, including public transport, such as buses; transport provided by the institution; private transport provided by third parties, some of which is paid for by the users themselves; their vehicles; and also by walking. However, the type of transport they use depends on their socioeconomic conditions and degree of autonomy.

(…) Sometimes, I come here on a small public bus, that passes through my area, and sometimes it feels like a swing because of the cobblestones. It is horrible; I would rather take the other bus because I get off at São Roque, and I would rather take that little thing that shakes all over the place (…)

(E2, woman, 79 years old)

(…) My daughter, a teacher at the Falcão school, brings me here in the morning and takes me in the afternoon. (…)

(E4, female, 85 years old)

(…) I often walk from here to my daughter’s street. So, I am used to walk a lot. Sometimes I go by car because I still drive (…)

(E8, man, 91 years old)

(…) I usually come in the centre’s van. I go to Soutelo and catch the van there. The money would not be enough if the van picked me up at home. I pay 60€ here, if I had to pay for transport, the money would not be enough (…)

(E5, male, 65 years old)

### 3.2. Autonomy to Move Around

About participants’ autonomy in getting around the area, some users can move around the area independently, using public transport and carrying out everyday activities. However, some depend on family members to help them in this process since they have limitations due to health complications. In other words, the participant’s health condition influences their autonomy to move around the area.

(…) I go to the pharmacy; I go to the bank to see my account statement and withdraw money. Yes, I do not walk downtown; it is far, and I can’t. However, once needed, I take the bus to the doctor (…)

(E16, female, 75 years old)

(…) only when my daughter can’t pick me up, I take the bus. But I manage fine on the bus (…)

(E4, woman, 85 years old)

(…) No, not anymore. I have to ask my children. Because, as I told you, I had a stroke and this is going very slowly (…)

(E6, woman, 77 years old)

4. Urban space perceptions

Another theme explored was the urban space of São de Roque de Lameira. Regarding participants’ perceptions of territory, it was possible to identify that their perspective of the area is linked to a vision of what the neighbourhood was like in the past. In other words, their perceptions are built on comparing how the neighbourhood used to be and how it is today. When making this comparison, users say they do not see any positive changes in the neighbourhood but rather an increase in structural and social problems. They attribute this stagnation and deterioration to the absence of public authorities.

(…) Everything is the same as it was. No one has done anything. The mayor does nothing (…)

(E6, female, 77 years old)

(…) It is not going to get any better than it is. Because in a few years, it will be the same (…)

(E7, male, 81 years old)

(…) I have lived here since I got married when I was 25. I liked it better the way it was. Now there are no neighbours like there used to be, it is all full of people and drugs. The houses are all empty (…)

(E9, woman, 91 years old)

5. Identified territorial challenges

Several difficulties were identified in the area, particularly concerning pedestrian pathways, which were in poor condition. The size and layout of the pavement, often narrow and uneven, also posed challenges to participants’ mobility within the neighbourhood. Another problem highlighted was the deterioration of the surroundings of the Community Centre. The participants mentioned the need for a refurbishment in this area so that they could make more use of the space, especially in the summer. In other words, the difficulties perceived by participants in the area are related to structural problems (e.g., the lack of urban furniture) in the urban space and the social context in which they live.

(…) The pavements are narrow, I walk slowly, and they are all bumpy. They should tar everything. Some are so small that they are smaller than the width of this table. They’re sloping (…)

(E5, male, 65 years old)

(…) It is more or less, but I cannot explain it either. The pavements are not in good condition. Some are higher, some lower. Full of loose stones, you fall, and nobody cares. (…)

(E10, woman, 86 years old)

(…) Everything is degraded surrounding this centre, so it should be fixed. Because then we could go outside more in the summer (…)

(E4, woman, 85 years old)

6. Difficulties faced by Illnesses

Concerning the difficulties users face in their illnesses, it was possible to recognise that some participants have mobility limitations, some acquired at a young age, and others developed over the years. These participants mention that these limitations make it difficult for them to move around, as they feel pain or get tired when they try to get around.

(…) I have been suffering from my prostate for many years, and I have been medicated for a long time. Also, back pain affects walking straight away. I get more tired (…)

(E8, male, 91 years old)

(…) Difficulty walking… I have a bad leg. I find it hard to walk. (…) I limit myself to my possibilities (…)

(E6, female, 77 years old)

(…) Apart from my wrist, which is annoying, it is just my bone problems that affect my mobility (…)

(E3, woman, 92 years old)

(…) I have had two falls because of my disability, meningitis. And then I fell in the street (…)

(E16, woman, 75 years old)

7. The role of public administration

Concerning the role of public administration in improving the territory, suggestions were made for revitalising the infrastructure of housing and public spaces. These include restoring public housing that is empty and dilapidated. Some participants say restoring these houses is important for reinvigorating the São Roque da Lameira neighbourhood. Another suggestion for improvement is to revitalise the garden located in the market area, to recapture what this area was like in the past. Some suggest that the public administration build a park in São Roque da Lameira to benefit the health and well-being of the neighbourhood’s residents.

(…) Restore all those abandoned houses out there. Many are privately owned, but there must also be state-owned ones. They are completely neglected and give the appearance of abandonment. If you look down the street in São Roque da Lameira, you will see the abandoned houses. It would bring more life to the area (…)

(E8, male, 81 years old)

(…) There, in the market area, we have a little garden. They could fix it and put it back the way it was before. Now it’s all destroyed (…)

(E6, woman, 71 years old)

(…) Make a park, at least for people to have fun there and spend a bit of time. Because even now, with the weather, autumn and winter, you cannot go outside. But in summer, at least, it is healthier (…)

(E15, female, 81 years old)

8. Suggestions for urban improvement

Concerning the suggestions for improvement, issues, such as afforesting the town to create an environment that provides thermal comfort for the population, were highlighted, as well as structural improvements to public roads and pedestrian routes. Other suggestions were made for the improvement of the surroundings of the Corujeira community centre.

(…) Cities look good with trees and plants. Shade is good for everything, even the climate. Trees help make the climate cooler. There is a lot to improve here, starting with better pavements, taking advantage of empty spaces to put some plants (…)

(E8, male, 91 years old)

(…) There should be better streets and even pavements for people to walk on. If these things were fixed up, it would be good (…)

(E10, woman, 86 years old)

(…) They should fix the surrounding streets. Moreover, a ramp should be put in here for the cars to enter. (…)

(E13, woman, 79 years old)

(II) ‘Go-along’ interviews

Sample Description

**Figure 3:**
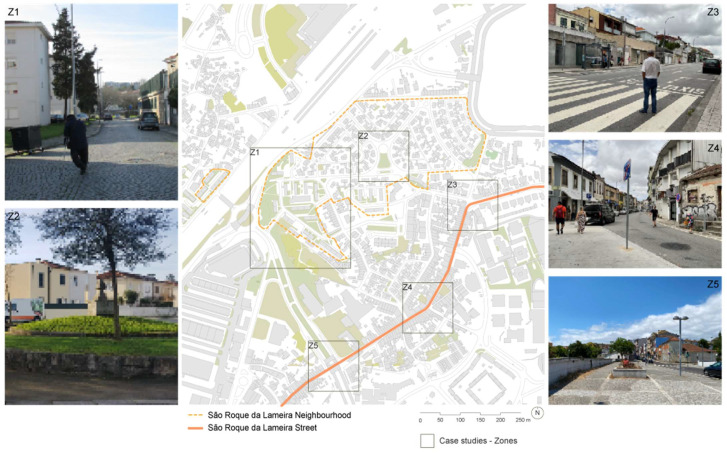
Approach locations for the ‘Go-along’ methodology. The five rectangles indicate the five areas in which the participants were approached and where, consequently, they responded to the interview. Source: CMP, © CAOP.

The ‘Go-along’ method revealed challenges and opportunities in the relationship between the older population and the public space, as the data collected pointed to structural barriers, various climatic impacts on their lives and the need for urban interventions to help make the city more accessible, inviting, and inclusive for older people. A total of 36 people with an average age of 73.9 ± 6.9 years were interviewed (Table 4). The highest concentration was in Zone 5 (13 participants), followed by Zone 3 (7), Zone 1 (6), Zone 2 (5), and Zone 4 (4). Only one participant’s location was not recorded (Figure 3).

Reading the profile of respondents indicates a group predominantly of people over the age of 66 (31 participants), with 6 of them over the age of 80. In addition to the age distribution, the gender imbalance stands out, with 22 men and 14 women.

The majority of participants (*n* = 31) are retired or receive a pension, while 4 declared themselves unemployed. The housing pattern reveals that 26 participants live with others (either family members or other individuals), 9 live alone, and 1 did not provide an answer.

9. Relationship with the Public Space, belonging and well-being

**Figure 4:**
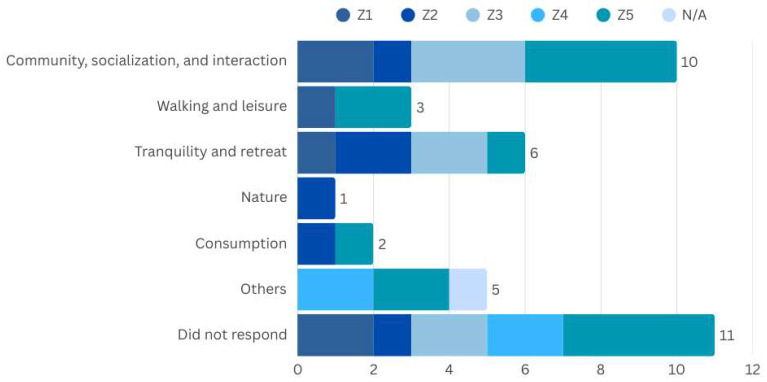
“What do you like most about this place/space (e.g., meeting friends)”? This chart illustrates the respondents’ preferences concerning the study area, indicating the corresponding approach areas to which each respondent is associated, as defined in Figure 3.

The majority of respondents prefer open public spaces, especially for daily activities during the day, as well as for leisure and socialising, with the majority carrying out these activities daily. It is important to note that the responses also mention tranquillity and seclusion as positive aspects of these places, which is in line with the general perception of the participants (Figure 4). When asked about their overall opinion of the space, 25 people rated it as good, very good, or excellent.

**Figure 5:**
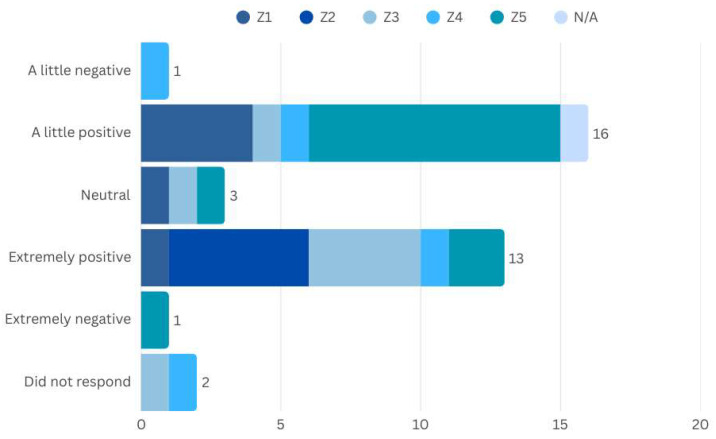
‘What do you feel when you’re in this place/space?’ This chart illustrates the respondents’ feelings concerning the place where they are being interviewed.

However, structural barriers, such as lack of accessibility, inadequate maintenance and issues related to insecurity make it difficult to stay in these places. Zone 5 stands out as one of the most challenging areas, especially in terms of infrastructure, negatively impacting the experience of belonging and well-being. In this context, 16 people expressed negative feelings towards the spaces in the region, 9 of them residents of Zone 5 itself (Figure 5).

Of the participants, 23 have lived in the area for more than 18 years, and 7 have lived there for more than 60 years. This factor indicates a temporal and emotional connection with the area, but it could also suggest an ageing population.

Most of the respondents indicated their habit of meeting in nearby areas, such as cafés, bakeries and markets, reinforcing the importance of these spaces in their daily lives and use of the city. Praça da Corujeira, Largo Valverde, and Parque São Roque are the green places most mentioned among the places the participants most like to visit.

10. Perception of the Neighbourhood and Feeling of Belonging

The perception of the neighbourhood is very good, with 15 participants reporting a positive assessment, 14 being extremely positive, while 4 were neutral, and 2 reported a slightly negative perception. The feeling of belonging seems to be related to the affective memory of the area. Many participants compare the neighbourhood to the past, mentioning an increase in structural and social degradation. However, some perceive improvements in the area, possibly related to recent urban interventions or population growth in the zone. This group, however, may not associate their mobility limitations with adverse environmental conditions, which highlights the need for greater awareness of the impact of infrastructure on local quality of life.

11. Comfort, Safety and Conservation of Public Space

Safety in public spaces is a central concern. The majority of participants (26) feel safe during the day, while 8 consider themselves partially safe, and only 1 reported feeling totally unsafe. The lack of adequate lighting and the presence of deserted spaces were cited as factors that increase the feeling of insecurity. The need to reinforce public lighting and improve urban infrastructure emerges as a priority to make spaces more welcoming and safer.

Conservation of public spaces was also mentioned. Of those interviewed, 24 considered that the pavement was not well maintained, while 12 believed that they were in good condition. Zone 5 was highlighted as the most challenging, which can be related to the lack of maintenance and the proximity of the highway, which increases the speed of vehicles and the diversity of dynamics in the street, making it less friendly for the older population.

12. Well-being in Hot and Windy Weather

Of those interviewed, 21 said they prefer to stay indoors on hot days, while 9 look for green areas or beaches. The lack of shade in public spaces makes it difficult to stay outdoors, as excessive heat is a determining factor in the decision to stay in public spaces. The absence of benches and covered areas exacerbates this situation, making the spaces less inviting for older adults.

13. Accessibility

Accessibility is a point that can be interpreted in some approaches, where more directly, 34 of the 36 participants get around on foot, but many also rely on public transport (9) and their own vehicles (3) even when travelling in very close proximity to the area. Uneven pavement and a lack of ramps were identified as barriers to mobility, especially in Zone 5 and Zone 1. Public transport, meanwhile, was considered uncomfortable due to the shakiness of the vehicles and the difficulty of accessing certain areas.

14. Amenities, Furniture and Urban Facilities

The lack of nearby urban facilities has an impact on social interaction and well-being. The frequency of cafés (8 responses) may be related to the uninviting spaces, even though there was a choice of squares and gardens (3 responses), the low diversity of options indicates the need for more leisure and socialising spaces focused on this public. The lack of benches with backrests, covered areas and street furniture in general reduces attractiveness and discourages older people from staying in the city.

15. Improvement Suggestions for the Territory

During the interviews, one of the main recommendations was the increase in tree planting in urban areas. This suggestion may be related to the importance given to the neighbourhood squares and gardens (10), which are also frequently mentioned as meeting points and places of social interaction, where people can feel good or enjoy shaded areas. This measure aims not only to provide thermal comfort but also to reduce the impacts of heat islands, creating a more pleasant and healthy environment for residents. Another recurring demand was the creation of resting areas equipped with benches and sheltered spots. There are reports of difficulty finding suitable spaces for breaks during daily walks. The creation of shaded areas with ergonomic furniture would be an ideal solution to promote the well-being of these people.

**Figure 6:**
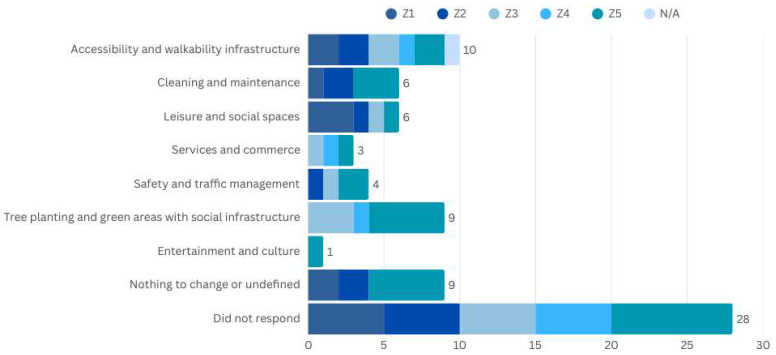
What would you change in this place/space (e.g., relocate some streetlights, trash bins…) and what would you like to see in this place/space (e.g., more green areas, more water points…)? This chart illustrates the changes respondents would like to see in the study area.

The infrastructure of sidewalks was also highlighted as a critical point. The installation of ramps and the improvement of pavement contributed to better walking conditions. The revitalisation of common spaces, such as squares, emerged as an important suggestion that contributed to spaces for socialising and refuge in nearby zones. Additionally, the creation of new urban parks, especially in areas with a deficit of green spaces, was highlighted, considering the importance of existing spaces mentioned. Expanding green areas benefits the physical and mental health of residents, offering leisure spaces within the city. Improvement of public lighting is also suggested to make urban spaces more inviting and safer, especially at night. Lastly, the expansion of hydration points and accessible public restrooms was identified as an important need. The presence of proper infrastructure, such as drinking fountains and accessible restrooms, ensures greater comfort and autonomy for older people, particularly during outdoor activities (Figure 6).

(III) User observations

16. Use and Users on-site observations

User observations in the São Roque da Lameira neighbourhood reveal that the use of these spaces is strongly influenced by individuals’ daily routines, social dynamics, and personal preferences. Beyond the usual groups of children playing, teenagers, and young adults, the older demographic is particularly noticeable. These individuals are often seen as couples taking leisurely walks, older women strolling with their grandchildren (who, due to limited access to daycare, rely on family support during work hours), and older people walking their dogs for exercise and companionship. Additionally, a group of older women often gathers for evening chats, fostering a sense of community and social bonding. The neighbourhood’s public spaces serve as important social hubs, where residents and non-residents alike can interact. Many people use these spaces as shortcuts, creating informal paths through the green areas, demonstrating a natural flow between private and public life. This blending of functions highlights the multifaceted role these spaces play in facilitating not only recreational activities but also essential daily routines, social interactions, and even moments of rest.

User observations in São Roque da Lameira Street revealed that most pedestrian movements are driven by proximity routines and needs, such as errands, like buying fresh bread or grocery shopping. For older adults, these activities often involve short round-trip routes and proximity circuits, where they take one street in one direction and return via a different route, blending daily chores with a leisurely stroll. The bus stops in this area, consistently crowded and quickly refilled after each bus departure, highlight the lack of adequate seating and shading. Older adults, in particular, often struggle to find places to rest along their routes, frequently stopping to catch their breath. With no dedicated rest points or seating furniture available, they are forced to rely on signposts for support or sit on low walls, reflecting the need for better infrastructure to accommodate the needs of pedestrians, especially older adults.

**Figure 7:**
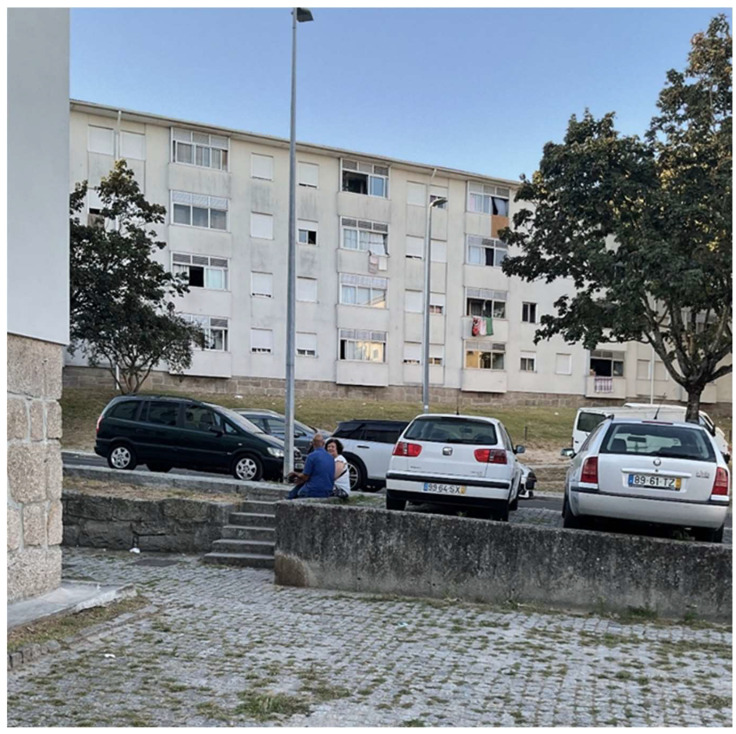
Examples of preferred meeting and socialising places. Source: CMP, © CAOP.

Thus, the user observations allowed for mapping trajectories and preferred gathering locations of the different age groups, yet paying special attention to the behaviour of older adults (Figure 7). This study revealed favourite concentration places in these areas, in particular close to cafes, bakeries and grocery shops. It also exposed the difficulties in using the public space, which were aggravated in the heat periods, such as: the configuration inadequacy of busy bus stops and lack of seating spaces, which requires many older adults to stand while waiting or to sit on pavement steps and surrounding shop entrances; an absence of shadows and the exposure to direct sunlight in waiting areas or the search for shadows even if more uncomfortable, such as seating in low walls or staircases; and several older adults were observed stopping along the trajectories to catch their breath, without a proper resting area, just holding on to a wall or post. These observations indicate that special attention should be paid to designing responsive public spaces that may be more inclusive and age-friendly, taking into account different rhythms, physical capacities, and sociability patterns.

(IV) Emotional mapping

Sample Description

This study, as shown in Table 5, had a sample of nine participants, six females and three males, with an average age of 76.7 ± 7.5 years. Concerning the participants’ level of education, the majority had completed the 4° grade. Regarding their living conditions, it was possible to identify seven participants who lived alone, and two who lived with their children and grandchildren.

This session revealed significant barriers, including uneven pavement, a lack of seating, and inadequate infrastructure for people with reduced mobility. Positive aspects, such as well-maintained buildings, green spaces, and essential services, were also highlighted. Participants provided valuable suggestions for improving public spaces, emphasising the need for better accessibility, comfort, and safety. These findings offer critical insights for urban planning initiatives aimed at creating age-friendly environments that enhance the quality of life for older adults in the context of climate change adaptation. Table 6 summarises the key findings from the emotional mapping activity, organised by zone. Each row corresponds to a specific location within the study area, detailing the barriers identified by participants (e.g., uneven pavement, lack of seating), the facilitators that enhance the space (e.g., well-maintained buildings, green areas), and the improvement suggestions proposed by participants (e.g., installing benches, repairing pavement).

**Figure 8:**
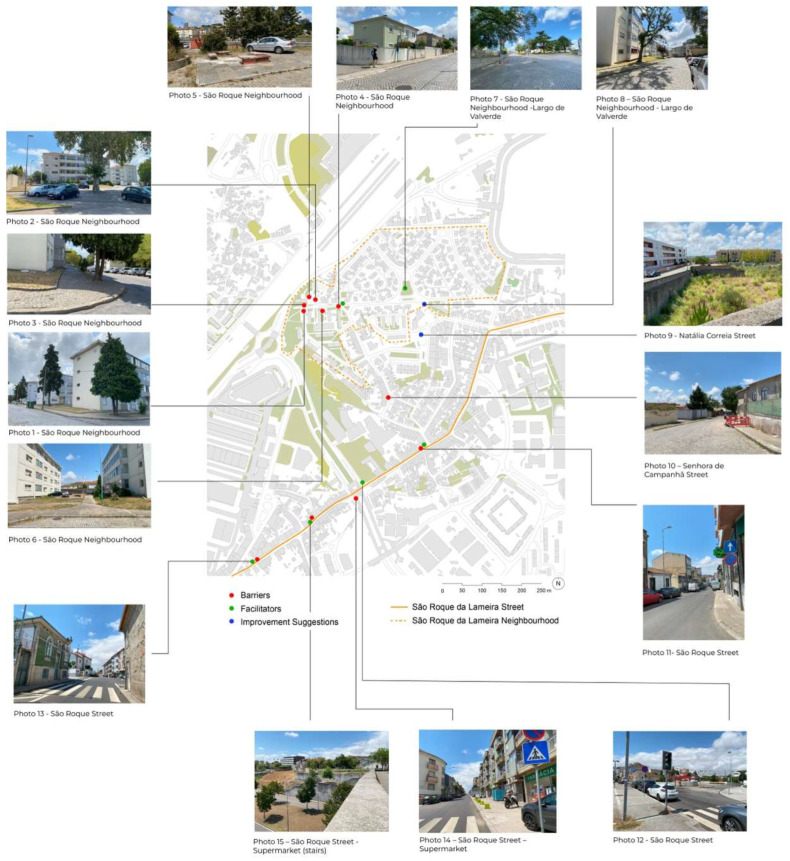
Emotional mapping results visualised on a spatial map. This figure illustrates the areas examined during the emotional mapping activity. It includes a photograph of each location, alongside markers indicating barriers (in red), facilitators (in green), and suggestions for improvement (in blue). Source: CMP, © CAOP.

To represent the findings graphically, a map of the study area was created, Figure 8, with colour-coded points indicating barriers (red), facilitators (green), and improvement suggestions (blue) based on participants’ responses during the emotional mapping activity.

## 4. Discussion

In this paper, adopting a *qualitative phenomenological approach*, four different participatory methods—individual semi-structured in-depth interviews (I), ‘go-along’ interviews (II), user observations (III), and emotional mapping (IV)—were used to explore older adults’ perceptions of climate change and its impact on health, as well as their daily experiences with health and mobility challenges in *São Roque da Lameira*, a vulnerable urban area in Porto, Portugal. The primary aim of this study was to gather insights that will inform future urban design and planning in this zone. This approach was designed to capture and interpret individuals’ lived experiences of the spaces and phenomena they inhabit and each method was carefully selected to deepen the understanding of participants’ subjective and embodied experiences of their. The findings offer essential contributions not only to understand the specific needs of this population but also to reinforce the importance of shaping inclusive and responsive urban design strategies for ageing populations.

Each methodology differed in terms of participant recruitment, sample size, level of participatory engagement, duration, complexity, and data collection approach (Table 1 and Table 7).

Regarding participant recruitment, gender distribution varied across methods due to both design and behavioural patterns. The individual interviews and emotional mapping had more women (12 out of 16 and 6 out of 9, respectively). An explanation for this can be that women show a greater willingness to share personal experiences and engage in reflective discussions [33]. Additionally, women frequent daycare centres to a greater extent than men, facilitating their recruitment for these methods [34]. Conversely, the ‘go-along’ method had a male-dominated sample (22 out of 36), possibly because men tend to maintain higher outdoor mobility, feel more comfortable in public spaces, and are more receptive to being approached for participation in such studies [35]. Women, on the other hand, deal with time constraints due to the polygonal mobility they mostly engage in, coupled with the need to balance everyday tasks, such as caring for children or dependent individuals. This can reduce their availability to participate in more spontaneous and external methods, such as the ‘go-along’ [36]. These differences highlight how gendered behaviours shape research participation. Finally, based on the tested hypotheses, recruitment was more challenging for the ‘go-along’ interviews due to the outdoor setting, lack of intermediaries, gender, and weather conditions, as people were more often in cafés and restaurants than on the streets, which made the thematic analysis more difficult. In contrast, open-ended interviews and emotional mapping were facilitated by contacts at daycare centres.

The different methods varied in the role of the participant, ranging from indirect participation to consultative and more active engagement. These variations reflect the spectrum of participation, from passive observation to active involvement. Depending on the context, adjustments are always necessary to make the results more accurate and representative in real-world applications. Emotional mapping was the only method used in this study that involved a collective format. This group dynamic fostered shared reflection, as participants were often reminded of experiences by others’ comments, enriching the discussion. It also allowed for the observation of social dynamics, such as points of consensus and disagreement, providing insights that individual methods may not reveal. Additionally, this format proved time-efficient and had the potential to strengthen participants’ sense of belonging and the value of their contributions. However, group-based approaches also come with limitations, including the risk of social pressure and self-censorship, which may inhibit honest expression. Unequal levels of participation can emerge, and individual perspectives may be explored with less depth compared to one-on-one methods.

The duration and logistical requirements of the four participatory methods varied significantly, reflecting their distinct approaches and the complexity of their proceedings. The open-ended interviews required the most time-intensive commitment, with each interview lasting 60 min. While this method provided in-depth insights, its extended timeframe may limit its feasibility in projects with tight schedules. In contrast, the ‘go-along’ method was more time-efficient, with each session lasting approximately 15 min. This approach enabled real-time observations but potentially limited response depth. The user observations involved six visits over one month (August 2024), with each session lasting between 3.0 and 3.5 h (summing several 30-min observations). Although this method provided rich contextual data, its reliance on extended observation periods may pose challenges in terms of researcher availability and participant consistency. Finally, the emotional mapping activity was conducted in a single 60-min session, making it the most time-efficient method. This approach was particularly suitable for older adults with limited mobility, as it eliminated the need for physical movement. However, its condensed format may have restricted the breadth of discussion compared to longer methods. Overall, the choice of methodology should balance the depth of data required with the practical constraints of time and participant availability, and the complementarity of perspectives allowed by each methodology proved valuable.

The data collection across the four methods varied in duration, setting, and degree of researcher involvement. Open-ended interviews and emotional mapping relied on structured indoor sessions, with the former involving multiple visits and audio recordings, while the latter was a single-session activity captured exclusively through field notes. In contrast, ‘go-along’ interviews and use observations were conducted in outdoor urban settings, with the former integrating real-time participant narratives during movement and the latter employing structured mapping of behaviours and spatial interactions. While the ‘go-along’ method emphasised direct engagement with the environment, use observations recorded patterns without direct participant input.

The analysis of data from the four participatory methods revealed common themes across the different methods. These included accessibility, safety and comfort in the area’s public spaces. In addition, issues, such as uneven pavement, lack of seating in squares, inadequate infrastructure for people with reduced mobility, and poor maintenance of public areas, were consistently highlighted. The unevenness of the pavement and the lack of ramps reinforce the accessibility challenges for older people, limiting their mobility independence and increasing the risk of falls in public spaces.

Based on these perceptions and from a problem-solving perspective, suggestions were made for improving the area. Ideas included designing an urban project to better accommodate older people, creating more green spaces in and around the neighbourhood, and improving the infrastructure of pedestrian routes, such as building wider pavement and easier crossings for pedestrians, including restrooms and drinking fountains to allow for more time in public space, as well as improving the condition of the city’s public transport system. These shared concerns reflect the current conditions of the territory but also point to the need to think about urban space that is more inclusive for older people (for instance, inadequate infrastructure reduces urban inclusiveness, limiting opportunities for social interaction—this is particularly significant given that some participants lived alone, a factor that may contribute to feelings of loneliness and a reduced use of public spaces). Similar concerns have been documented in age-sensitive urban design studies in cities, like Buffel et al. [37], Drescher and Skoyles [38], where older residents also identified issues, such as poor walkability, lack of greenery, and fear of crime, as key deterrents to active public life. Zumelzu et al. [39] also highlight the crucial role of age-friendly urban infrastructure, such as accessible pavement, green spaces, and seating areas, in promoting health and well-being among the older population. These findings point to the universality of certain urban vulnerabilities, even if they are locally shaped. Another notable similarity that emerged from both the interviews and the go-along method was the tendency of older participants to perceive their surroundings through a comparative lens, contrasting the present state of the neighbourhood with how it was in the past. Our findings are aligned with the results from previous studies, which also indicate that older adults in disadvantaged urban areas perceive infrastructural decay, insufficient shade, and public safety as key concerns [40,41]. However, few of those studies employed real-time participatory tools, making our findings particularly relevant for advancing methodological innovation in the field.

Although similar themes emerged, each method allowed unique insights. Concerning individual open-ended interviews, it was possible to identify that this type of strategy explored users’ individual experiences and perceptions of the territory, as well as the impacts of urban degradation on their lives. Notably, these interviews were the only method that enabled us to infer older adults’ views on climate change and its perceived implications for their health. Interview participants expressed concern about rising temperatures and weather instability, which they associated with worsening symptoms of existing health conditions and a decline in overall quality of life. In contrast, the ‘go-along’ interviews allowed researchers to observe participants’ real-time interactions with their surroundings. This method made it possible to identify barriers, affordances, and emotional responses. For example, when participants reflected on desired changes to their surroundings, they often looked around, engaging directly with the space and its elements. As they walked and responded to the questions, they became more aware of the challenges they faced during mobility, such as the lack of space on pavement. Regarding the user observations, this approach provided an objective account of daily activities, usage patterns, and unspoken behaviours in public space, in this sense the researchers were able to capture nuanced interactions and habitual practices that might not be revealed through direct questioning, offering a deeper understanding of how individuals engage with their environment, without realising their emotional connection or sense of belonging to the place. It is understood that this method is particularly valuable in the study of accessibility, inclusion, and usability of public spaces. On the other hand, the emotional mapping encouraged participants to associate specific places in the neighbourhood with feelings, offering a spatial representation of the positive aspects but also of the challenges, shortcomings and transformations all in a safe and comfortable place for the participants.

Regarding challenges, the open-ended interviews were dependent on participants’ selective memory and difficulty verbalising subjective experiences, which compromised the depth of the data collected. Furthermore, the limited application to a single centre restricted the diversity of perceptions, suggesting the need to expand to other locations. The ‘go-along’ interviews faced challenges related to the reduced mobility of some participants, the excessive number of questions, and the cold weather, which contrasted with the central theme of heat. These factors impacted the systematisation of the results, the duration of the journeys, and participants’ willingness to provide detailed responses. Many had never reflected on the topic, making their responses more difficult. This emphasises the need to adapt the routes, revise the survey structure, and conduct the methodology in warmer periods. The observation of space use recorded spontaneous behaviours and movement patterns but lacked subjective context, which is essential for understanding the motivations behind these actions. The emotional mapping had a small sample size and difficulties in categorising the data due to the subjectivity of the responses, which compromised the representativeness of the perceptions collected. Expanding the sample, increasing the listening time, and diversifying the spaces analysed are key measures to achieve more focused and representative results.

Combining four distinct methods in this study provided a deeper understanding of the challenges and opportunities experienced by older adults in the territory (Table 7). While all methods yielded valuable insights, the ‘go-along’ interviews and emotional mapping were particularly effective in capturing real-time emotional and spatial responses—elements that traditional interviews alone may overlook. This highlights the value of participatory techniques and supports the use of mixed-methods approaches in urban studies. These hybrid strategies are increasingly recognised as best practices for involving marginalised populations in climate adaptation planning, as reflected in recent frameworks by the Urban Ageing Consortium and UN-Habitat [42]. Overall, the study illustrates how combining narrative, observational, spatial, and affective methods enables a multidimensional understanding of ageing and climate adaptation in urban contexts.

However, the study has some limitations. Despite the rich insights generated, the study’s scope is limited to one urban context. While São Roque da Lameira shares characteristics with other ageing and under-resourced areas, caution should be exercised in generalising the findings. Future research should replicate this participatory approach in different geographic and socioeconomic contexts to assess its transferability. In addition, a limiting factor was the total sample size, which, although diverse in terms of gender and socioeconomic background, could have been larger, compromising generalisability and also a certain degree of subjectivity in identifying a theme and the challenge of generalising results beyond the population studied. Future research, therefore, should consider expanding the sample and integrating digital tools to collect real-time data.

For future research, when using a similar framework with different participatory methods, some aspects need to be taken into consideration. The sample must be defined to ensure greater diversity, and the methods should be adapted to local specifics. To achieve this, it is recommended [43] to expand the data collection points, categorise participants, and adjust surveys and approaches according to their profiles, differentiating those who live in the territory from those who only visit it. Expanding the network of partners in the application of methods, including community leaders, is also essential. Other adaptations may include conducting studies in warmer months, adjusting routes according to mobility conditions and research objectives. These changes will not only improve the quality of the data collected but also make the methods more representative of the participants’ reality and territory.

This study suggests that participatory methods can inform urban planning policies focused on ageing-in-place strategies, particularly in socioeconomically vulnerable neighbourhoods. As argued by Pelling et al. [44], participatory planning not only strengthens resilience but ensures that interventions are grounded in local knowledge and tailored to community-specific vulnerabilities. This resonates with Marston and Hoof [45], who argue that urban interventions that incorporate emotional and social dimensions, not just physical ones, are more likely to support the subjective well-being of older adults.

By foregrounding the voices and perspectives of older adults, this study also engages with broader international agendas that prioritise inclusive and participatory approaches to urban and climate planning, such as the global movement towards age-friendly cities [46] and climate-resilient urban planning [47]. Through the use of multiple participatory methods to understand how urban infrastructure and climate stressors intersect in older adults’ lives, this study is consistent with the World Health Organisation’s framework for age-friendly cities and communities [48], which stresses the role of building environments in enabling active ageing, and with the IPCC’s recognition of social vulnerability in urban adaptation planning [49]. In fact, Ong et al. [34] emphasise that participatory governance can improve both the democratic legitimacy and the effectiveness of climate action, particularly when rooted in local knowledge and everyday practices. This represents a shift from extractive consultation to co-production and co-design processes [50], facilitating more sustainable and equitable urban transformations.

Ultimately, our findings highlight the urgent need to integrate participatory methodologies into urban planning to ensure older adults’ voices are included in climate adaptation strategies. In doing so, policymakers and urban planners can create environments that support active ageing and mitigate climate-related risks for this population.

## 5. Conclusions

This study provides valuable insights into how older adults experience and navigate urban vulnerability, particularly in the context of climate change and health, within São Roque da Lameira. It yielded critical insights for both research and practice in age-inclusive and responsive urban planning. Through the use of four participatory methods, it was possible to uncover not only recurring concerns, such as accessibility, safety, and infrastructural neglect, but also emotional connections to place and nuanced behaviours that often remain unspoken. Each method illuminated distinct dimensions of urban life, reinforcing the value of a plural, context-sensitive approach to participatory research with older populations.

This multidimensional methodology can be useful to urban planners and practitioners, including the adoption of: 1) combined methods to balance depth and breadth, 2) adaptive protocols to account for weather and mobility constraints, and 3) tools that rely on visual prompts, tactile objects, and participatory mapping to support engagement. The positive reception to participatory design proposals by older adults demonstrates how integrating diagnostic tools (identifying issues) with propositional techniques (generating solutions) enhances participatory outcomes. Moreover, participatory methods offer access to spatial, social, and emotional dimensions of urban life, often missed by conventional assessments. This ethnographic sensitivity fosters a deeper understanding of the cultural meanings and everyday practices that shape place attachment and vulnerability. It encourages respectful interventions that acknowledge the identity of people and the memory of places. By improving the quality of public spaces and reconfiguring, it becomes possible to promote climate adaptation without erasing the material and affective histories embedded in urban territories.

Future research should consider expanding the geographical scope, increasing sample diversity, and refining participatory tools to enhance representativeness and engagement. Ultimately, fostering inclusive and resilient urban environments requires not only listening to older adults but actively involving them in shaping the cities they inhabit.

## Figures and Tables

**Table 1:** Main aspects of the four participatory methods used.

Method	Objective	Duration	Required Materials	N° of Participants	Type of Participation
(I) Open-ended Interviews	Explore perceptions and experiences of climate change, mobility, and well-being	60 min per interview, conducted over 4 months (June-Oct 2024)	Interview guide, audio recorder, informed consent	16	Active participation, answering questions in a structured setting
(II) ‘Go-along’ Interviews	Understand how older adults perceive and interact with the urban environment in real-time	15 min per participant, conducted over 2 months (Jan-Feb 2025)	Interview guide, informed consent	36	It combines consultative and active participation, engaging participants in research without decision-making influence
(III) User observations	Analyse behavioural patterns, movement flows, and social interactions in public spaces	30 min of observation at each location (August 2024)	Photographs of locations, KoBoToolbox, hand-drawn sketches, GIS Field maps, field notes	Not applicable (passive observation)	Indirect/passive participation, no direct interaction with observed individuals
(IV) Emotional Mapping	Identify positive and negative perceptions of public spaces and suggest improvements	60 min, single session (Feb 2024)	Photographs of locations, red and green cards, informed consent	9	Active participation, expressing opinions on images and engaging in group discussions

**Table 2:** Sociodemographic characterisation of the interview participants.

Interviewee Code	Gender	Age	Education	Living Situation
P1	Female	72	4° grade	Living alone
P2	Female	79	3° grade	Living alone
P3	Female	92	3° grade	Living together
P4	Female	85	4° grade	Living together
P5	Male	65	3° grade (incomplete)	Living alone
P6	Female	77	4° grade	Living alone
P7	Male	81	4° grade	Living alone
P8	Male	91	10° grade	Living together
P9	Female	91	4° grade	Living alone
P10	Female	86	4° grade	Living together
P11	Male	67	4° grade	Living alone
P12	Female	91	4° grade	Living alone
P13	Female	79	11° grade	Living together
P14	Female	93	4° grade	Living alone
P15	Female	81	3° grade	Living alone
P16	Female	75	4° grade	Living alone

**Table 3:** Categories and subcategories created in the thematic analysis of interviews.

Categories and Subcategories	Description
1. Participants’ perceptions of climate change	Organises participants’ perceptions on climate change.
2. Challenges of adverse weather	Organises participants’ difficulties due to adverse weather conditions.
3. Urban mobility	Gathers insights on participants’ experiences and perceptions of urban mobility.
- 3.1. Autonomy to move around	Subcategory of Urban mobility, addressing participants’ ability to move independently.
- 3.2. Modes of transport	Subcategory detailing the types of transportation the participants use.
4. Urban space perceptions	Organises participants’ perception of the urban space.
5. Identified territorial challenges	Documents the difficulties participants identified in the territory.
6. Difficulties faced by Illnesses	Addresses participants’ health-related difficulties.
7. Role of public administration	Explores the role of public administration in territorial (or urban) improvement.
8. Suggestions for urban improvement	Collects participants ‘ suggestions for enhancing the urban territory.

**Table 4:** Results of the profile of respondents approached for the ‘Go-Along’ survey in the survey neighbourhoods.

PROFILE OF RESPONDENTS		
CATEGORY	CHARACTERISATION	TOTAL
Total Respondents	Respondents Over 65	36
Age	65–69	12
	70–74	8
	75–79	8
	80+	8
Gender	Female	14
	Male	22
Schooling	Cannot Read and Write	1
	4th Year Of School Or Less	12
	Up To 9th Grade	5
	9th Year Of School	5
	Secondary School	2
	Non-higher Technical Education	1
	Incomplete Higher Education	1
	Higher Education Completed	8
	Did Not Answer	1
Profession	Industry And Production	4
	Construction And Maintenance	10
	Services And Commerce	11
	Public Sector And Security Forces	5
	Education And Health	4
	Transport And Mobility	1
	Did Not Answer	1
Labour Situation	Retired/Pensioner	31
	Unemployed	4
	Self-employed	1
If You Live In The Neighbourhood	Yes	32
	No	4
Yes: How Long Have You Lived In The Neighbourhood?	Under 18	9
	18 To 30 Years Old	3
	31 To 50 Years Old	7
	51 To 60 Years Old	6
	Over 60	7
No: What Brings The Neighbourhood?	Visiting People	2
	Shopping	1
	Did Not Answer	1
If You Live Alone/Who You Live With	Wife/Husband (Even If Not Married)	15
	Wife/Husband and Child(ren)	4
	Child(ren) Only	3
	Lives Alone	9
	Parents (Father And/Or Mother) And Other Relatives	2
	Other	2
	Did Not Answer	1

**Table 5:** Sociodemographic characterisation of the emotional mapping activity participants.

PROFILE OF RESPONDENTS		
CATEGORY	CHARACTERISATION	TOTAL
Total Respondents	Respondents Over 65	9
Age	65–69	2
	70–74	1
	75–79	2
	Over 80	4
Gender	Female	6
	Male	3
Schooling	3rd Year of School	1
	4th Year of School	8
Living Situation	Living Alone	7
	Living with children	2
Labour Situation	Retired/Pensioner	7
	Permanent illness/incapacity	1
	Household/Family Care	1
Marital status	Divorced	1
	Widowed	5
	Single	3

**Table 6:** Results of the emotional mapping activity, organised by thematic categories (barriers, facilitators, and improvement suggestions) for each zone.

Zone	Barriers	Facilitators	Improvement Suggestions
Photo 1—São Roque Neighbourhood	Uneven pavement, holes, lack of seating places	Well-structured buildings	Install benches, repair pavement
Photo 2—São Roque Neighbourhood	Difficult parking, lack of benches	Trees, shaded areas, well-maintained buildings	Increase green area, add seating areas
Photo 3—São Roque Neighbourhood	Uneven pavement, lack of ramps for wheelchair users	-	Install ramps, repair pavement
Photo 4—São Roque Neighbourhood	Raised drainage covers, narrow pavement, inadequate bus stop shelter	Well-located bus stop	Widen pavement, install bus stop shelter, lower drainage covers
Photo 5—São Roque Neighbourhood	Untreated grass, uncomfortable stone benches, lack of maintenance	-	Install benches with backrests, maintain green spaces
Photo 6—São Roque Neighbourhood	Poorly maintained area, excessive concrete	-	Plant flowers and trees, increase cleaning frequency
Photo 7—São Roque Neighbourhood -Largo de Valverde	-	School, large garden, pleasant square	-
Photo 8—São Roque Neighbourhood—Largo de Valverde	Overgrown trees, uneven pavement	Refurbished apartments	Trimming trees, repair pavement
Photo 9—Natália Correia Street	Empty space	-	Build a green park or create social centre or playground
Photo 10—Senhora de Campanhã Street	Narrow pavement, unsuitable for people with special needs	-	Widen pavement, improve accessibility
Photo 11- São Roque Street	Poorly parked vehicles	Smooth tar	Enforce parking regulations
Photo 12—São Roque Street	Poorly located pedestrian crossing, broken pavement, lack of benches	Smooth tar	Relocate pedestrian crossing, repair pavement, install benches
Photo 13—São Roque Street	Narrow pavement, heavy traffic, dangerous intersection	Smooth tar	Reduce traffic, improve pedestrian safety
Photo 14—São Roque Street—Supermarket	-	Wide pavement, essential services (e.g., pharmacy)	-
Photo 15—São Roque Street—Supermarket (stairs)	Construction over water source, lack of external improvements	Matadouro redevelopment project, Oriental Park	Improve external area, consider environmental impact of projects

**Table 7:** Comparison and evaluation of the methods used. Consider a scale of 1 to 5, where 1 means ‘objective not achieved’ and 5 means ‘objective fully achieved’.

METHODOLOGY	TARGET	IMPLEMENTATION	CHALLENGES	LIMITATIONS	RECOMMENDATIONS FOR REAL-WORLD SITUATION
Individual interviews	Fully achieved	Partially achieved	Not achieved	Partially achieved	- Use observational methods to capture non-verbalized aspects. - Improve question formulation to facilitate memory recall and verbalization. - Implement visual aids (such as photos or videos) to encourage more detailed responses. - Expand the application sites to other Daycare Centers, for example.
	Understand older adults’ perceptions of climate change, health, mobility, and public space	Conducted with 16 participants at a Day Center, using open-ended questions	Selective memory challenge: Participants may not recall details of their past experiences accurately. Difficulty in verbalization: Some participants have trouble expressing subjective experiences.	Lack of direct spatial context (may not fully capture participants’ real-time interactions with urban spaces or the practical challenges they face daily). Reliance on memory and perception	
Go-along interviews	Partially achieved	Not achieved	Not achieved	Partially achieved	- Conduct the methodology during the summer or in warmer periods. - Adapt the routes based on participants’ mobility capabilities. - Expand the sample to ensure the inclusion of more women, people with reduced mobility, and disabilities. - Reformulate some open-ended questions. - Expand the outreach areas near local institutions.
	Record older adults’ perceptions of public space and their lived experiences (challenges and potentials)	36 interviews conducted while participants walked through the neighborhood	Mobility restrictions: Mobility difficulties limit the length of the routes. Cold weather: Cold weather hindered detailed assessment and reduced participation. Superficial responses: Questions not formulated in a targeted manner may lead to superficial responses.	Challenges related to limited mobility and cold weather, restricted the central objective of the expected results. Difficulties to map the trajectories enterviews.	
Emotional mapping	Partially achieved	Not achieved	Not achieved	Partially achieved	- Increase the sample size to ensure greater diversity of responses. - Combine with qualitative methods (interviews or observations) to gain more context. - Diversify the data with different types of spaces, such as public and private spaces frequented by older adults. - Expand partnerships with local institutions focused on older adults, which could be useful in increasing the sample size.
	Identify positive and negative aspects of the urban environment from the perspective of older adults	9 participants evaluated images of neighborhood spaces, indicating positive perceptions (green card) and negative perceptions (red card)	Subjectivity in responses: The interpretation of images may vary according to each participant’s personal experiences, making categorization difficult. Small sample size: The small sample size may not reflect the diversity of the elderly population.	Participants may struggle with the abstract nature of the task or feel uncomfortable expressing emotions, leading to incomplete data	
Observation of space use	Fully achieved	Partially achieved	Not achieved	Partially achieved	- Combine observations with interviews. - Conduct observations at different times and days of the week. - Integrate with other methodologies. - Strategic locations such as squares, public parks, and markets should be more frequently addressed, in addition to consulting senior citizen social centers.
	Map movement patterns, gathering areas, and challenges faced by older adults in public spaces	Observations in strategic spaces on hot days to identify movement patterns and space usage	Difficulty in capturing subjective perceptions: Observation alone may not be sufficient to understand the motivations and feelings of participants regarding the spaces. Influence of momentary events: Observation can be influenced by temporary factors, such as sporadic events that alter behavior patterns.	Observations requires long times observations	

## Data Availability

The original contributions presented in this study are included in the article. Further inquiries can be directed to the corresponding author.

## Appendix A

Guide for the Semi-Structured Interviews.

For the individual interviews, the following semi-structured guide was used:

“Part I—Identification and Characteristics of the Participant

- What is your name and age?
- Do you live alone or with your family? If not, in what type of living situation do you reside?
- How long have you lived here? Or how much time do you spend here?
- What is your level of education, what do you do, or what did you do before retirement?
- Do you consider that you have enough money to meet your needs? (None, Little, Moderately, or Quite a lot)

Part II—Perception of Climate Change and Its Impact on Health

As we mentioned, this interview aims to understand the negative effects of climate change on people’s lives. And what is that? Over recent years, with climate change, the climate/weather has become warmer, colder, drier, or rainier than before.

- Have you noticed these changes in the area where you live? In what way? How does it affect your day-to-day life? Which changes are the hardest to overcome/endure?
- Do you recall any situations where you felt physically unwell or experienced complications due to the weather, such as fainting, slipping because of rain, or losing balance due to wind?
- Have you ever felt sadder, more anxious, or confused because of the climate/weather?
- Have these changes in climate/weather ever prevented you from sleeping well?

Part III—Perception of Everyday Life in Urban Spaces

Now that we know you a little better, we would like to understand your daily routines and what you usually do.

- Where do you typically go during the day? Could you describe what a normal day looks like for you?
- What routes do you usually take? Do you take these routes alone, with others, on foot, by public transportation, or by private transportation?
- Do you use nearby public spaces? Are these spaces comfortable? Do you feel there’s a lack of something, such as shade, resting areas (benches), or water to refresh yourself?
- Do you have any problems or difficulties in your routines? Can you tell us more about them? Do you have trouble getting in or out of your home? Are the streets too steep to walk? Are bus stops sparse and far apart? Are public services in difficult-to-reach locations? Are sidewalks in poor condition?
- How do these difficulties affect your life? What would help you overcome these challenges? Do you use any strategies to deal with these challenges, such as choosing certain times to leave the house, opting for routes with more shade, or taking a taxi?

Part IV—Health and Mobility Issues

Focusing on your health and your ability to move from one place to another, whether on foot, by car, public transport, or another means of transportation, please tell us:

- What health issues do you currently have? How do these issues affect your mobility and quality of life?
- Do you think that the place where you live has any relation to your health and mobility problems? If yes, in what way? What strategies do you use to cope with this?

Part V—Closing

To conclude, considering all the health and mobility limitations we discussed, which are in some way a result of climate change, what strategies do you think local authorities should implement to improve your area and address these issues?”

## Appendix B

Guide for Conducting Semi-Structured Interviews in LimeSurvey Format.

**GENERAL INFORMATION**	- Date and time of questionnaire. - Identification of the name of the place/space. - Geolocation of the place/space. - Photographic record of the place/space.
**PARTICIPANTS**	- How old are you? - Do you live in the neighbourhood? - If yes, how long have you been living in the neighbourhood? - If not, how often do you come to the neighbourhood? Are you a worker, a regular visitor or a tourist/sporadic visitor?
**CHOICES**	- Do you like going to an open public space? - If yes, why and at what time? - If not, why not? - What is the most important or interesting place in São Roque da Lameira, and why? - Where is it located? - Can you think of any public spaces or places that have lost importance recently? - Where is it located? - If you had to arrange a meeting point, where would it be? - What do you like to do the most when away from home? - How often do you visit this place? - Why have you come to this place today? - How long do you expect to be here today?
**SENSE OF BELONGING AND WELL-BEING**	- How do you feel about this neighbourhood? - What do you feel when you are in this place/space? - What do you like the most about this place/space? Why do you like it? - What do you not like about this place/space? Why do you not like it? - Is there any noise, dust, odours or other types of pollution? - What is your general opinion of this place/space (in terms of design and long-term conservation)? - Do you feel welcome in this place/space? Why? - Does it have a good scale? Are there any landmarks? - Do you see people with special needs (parents with small children, people in wheelchairs or using walking sticks, people with visual or hearing impairments) walking/rolling through this area?
**COMFORT AND SAFETY**	- Do you feel safe, partially safe or not at all safe in the place/space? - Is this public space considered safe during the day and at night? - Are there people and activities at any time of the day? Why? - Does the lighting provide safety at night and a good atmosphere? - Are there places where it is not advisable to go? - What obstacles and difficulties do you encounter in this place/space? - Do you feel safe when walking on the pavement? Why? - Do you think people of different ages feel safe?
**STATE OF CONSERVATION OF PUBLIC SPACE**	- Do you think the pavement are well maintained? Why do you think so? - What do you think about the cleanliness of the streets? - Are there any vandalised elements around this place/space?
**PERCEPTION OF WELL-BEING IN HOT AND WINDY WEATHER**	- When it is very hot, where do you usually go to feel comfortable? - Do you think there is enough shade in this area? - Do you think this place has enough trees or green areas? - Do you think the benches, garden tables, and bus stops are protected from the sun, rain and wind? - Does the space work well when it is windy? Are there shelters against intense sun, rain or minor flooding? - When it is hot, which garden or square do you use the most? - On very hot days, which street is the most pleasant to walk along?
**ACCESSIBILITY**	- How do you get to this place/space? - Do you use any means of transport? - Do you think the public transport stops are sufficient and well located? - When you walk, which route do you use the most? - Is it easy to get around in this area? Are there any constraints? - Do you think there are enough pedestrian routes? - Do you think there are enough ramps for wheelchairs? - Would you like to see a network of cycle paths in the area?
**AMENITIES, FURNITURE, AND EQUIPMENT**	- Where do you usually go when you feel lonely and need to talk to someone? - Do you feel the need for space to socialise? - Do you think people can sit or stand in this space? - Do you think this space is suitable for physical activity? - Do you think there are enough playgrounds in the area? - Are there enough benches and garden tables? Are they well-located? - Do you think there is a need for sanitary facilities? - Are the waste recycling facilities properly used?
**IDEAS/SUGGESTIONS**	- What would you change about this place/space? - What would you like to see in this place/space?
**SOCIO-DEMOGRAPHIC CHARACTERISTICS OF THE PARTICIPANTS**	- What is your level of education? - What is/was your profession? - What is/was your work situation? - Where is/was your workplace? - Do you live alone? If you don’t live alone, who do you live with?
